# Depigmenting Effect of Catechins

**DOI:** 10.3390/molecules14114425

**Published:** 2009-11-04

**Authors:** Kazuomi Sato, Masaru Toriyama

**Affiliations:** 1 Department of Life Science, College of Agriculture, Tamagawa University, 6-1-1 Tamagawa-gakuen, Machida, Tokyo, 194-8610, Japan; 2 Department of Applied Biological Chemistry, Faculty of Agriculture, Shizuoka University, 836 Oya, Shizuoka, 422-8529, Japan; E-Mail: acmtori@agr.shizuoka.ac.jp (M.T.)

**Keywords:** catechin, gallic acid, melanogenesis, B16 melanoma, tyrosinase, α-MSH (α-melanocyte stimulating hormone)

## Abstract

The aim of the present work was to clarify the anti-melanogenic mechanism of the catechin group. In this study, we used (-)-epigallocatechin-3-gallate (EGCG), (-)-epigallocatechin (EGC), (-)-catechin (C), and gallic acid (GA). The catechin group inhibited melanin synthesis in B16 melanoma cells. To elucidate the anti-melanogenic mechanism of the catechin group, we performed Western blotting analysis for crucial melanogenic protein, namely tyrosinase. The catechin group inhibited tyrosinase expression. These results indicate that the catechin group is a candidate anti-melanogenic agent and that it might be effective in hyperpigmentation disorders.

## Introduction

Human skin pigmentation is caused by melanin synthesis in UV irradiated melanocytes. Tyrosinase is a key enzyme in melanin synthesis that can catalyze three different reactions: the hydroxylation of tyrosine to 3,4-dihydroxyphenylalanine (DOPA), the oxidation of DOPA to DOPAquinone and the oxidation of 5,6-dihydroxyindole (DHI) to indole-quinone [1]. In the absence of thiols, DOPAquinone changes to DOPAchrome and then to DHI or indole 5,6-quinone 2-carboxylic acid (DHICA). There are two other main enzymes in this melanogenic pathway: tyrosinase-related protein-2 (TRP-2; DOPAchrome tautomerase), which catalyzes the conversion of DOPAchrome to DHICA, and TRP-1 (DHICA oxidase), which catalyzes the oxidation of DHICA [2,3,4,5,6].

Investigation of the depigmentation mechanisms and clinical aspects of skin-whitening agents is very important. In addition, increased production and accumulation of melanin leads to many hyperpigmentation disorders such as melasma, postinflammatory pigmentation, solar lentigo, etc, which become prominent with aging. Many chemicals have been demonstrated to show inhibitory effects on melanogenesis through inhibition of the enzymatic activity of tyrosinase, but their effects on related gene expression, tyrosinase degradation and glycosylation, melanosome transfer, and cellular signaling regulation are also reported to control melanogenesis (reviewed by Solano *et al*. [7] and Briganti *et al*. [8]). 

**Figure 1:**
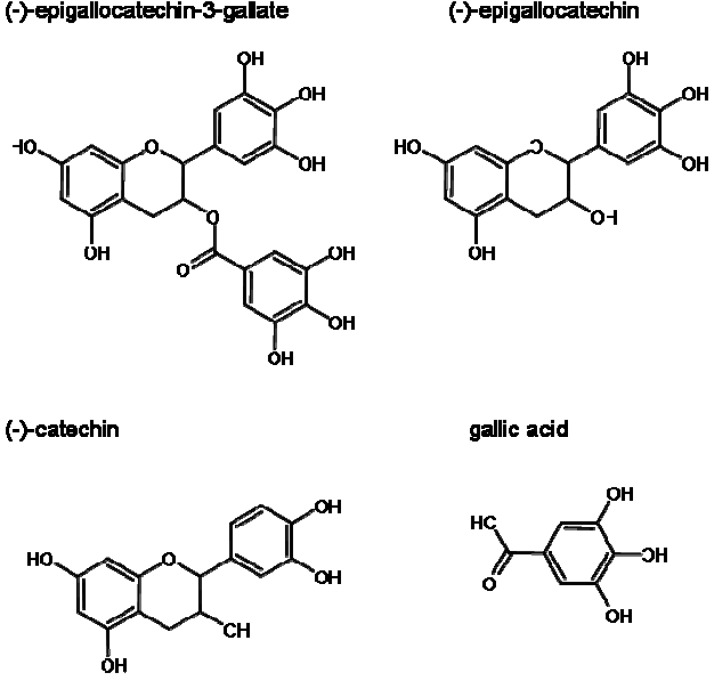
Chemical structures of catechins and gallic acid.

Green tea includes several polyphenolic compounds. Tea extract contains (-)-epigallocatechin-3-gallate (EGCG), (-)-epigallocatechin (EGC), (-)-catechin (C), (-)-gallocatechingallate (GCG) and (-)-epicatechingallate (ECG) [9]. Especially, EGCG is the most abundant in the tea extract and is the most active in the catechin group. Moreover, EGCG is the most well-studied catechin derivative. For example, Kim *et al*. reported that EGCG reduces melanin production in mouse melanoma cells [10]. However, depigmenting effects of other catechin derivatives are not well known. Therefore, in the present study, to compare the depigmenting effect of catechin derivatives, we investigated the melanogenesis inhibitory mechanisms of the catechins (EGCG, EGC and C) and gallic acid (Figure 1) in murine B16 melanoma cells, and we also discuss their effects on the expression of tyrosinase.

## Results and Discussion

**Figure 2:**
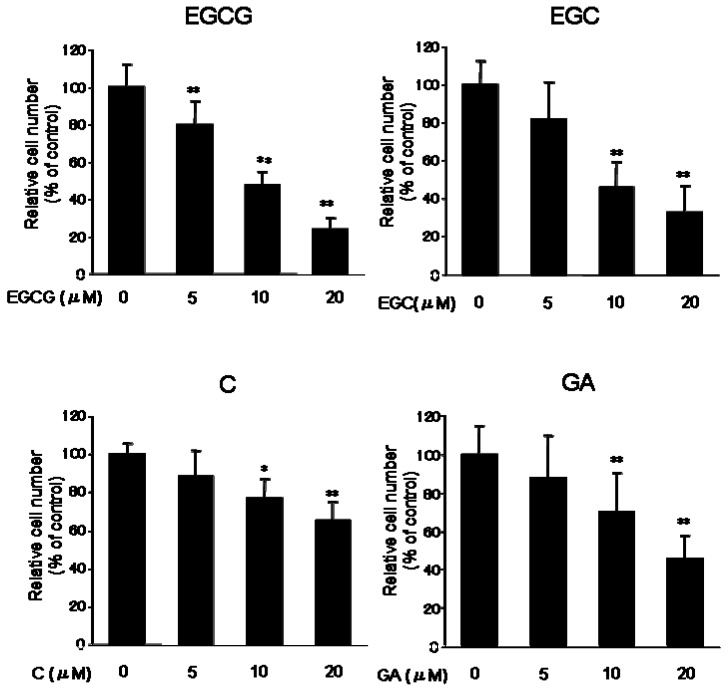
Effect of catechins (EGCG, EGC and C) and GA on the proliferation of B16 melanoma cells. B16 melanoma cells were cultured for 5 days with or without test substances and harvested as described in the Experimental. The number of cells was counted. Results are expressed as a percentage of control culture, and data are the means ±S.D. of at least three separate experiments. *P < 0.05 and **P < 0.01 versus control group.

In the present study, we have demonstrated the inhibitory mechanism of catechins in B16F1 murine melanoma cells. At first, to test the effect of the catechin group on cell proliferation, B16 melanoma cells were incubated with EGCG, EGC, C, and gallic acid (GA) (5, 10, 20 μM) for 5 days. All test substances inhibited cell proliferation (Figure 2). Especially, EGCG showed the strongest inhibition of cell proliferation (approximately, 48% at 10 μM, 24% at 20 μM) and some cells detached from the dishes. Several reports also indicate that EGCG shows growth arrest of lung, colon and ovarian cancer [11,12,13]. Mitsuhashi *et al*. reported that polyphenol (EGCG, EGC and GA) treatment inhibited cell proliferation in the human leukemia cell line K562 and the human embryonic kidney (HEK) 293T cell line [14]. 

**Figure 3:**
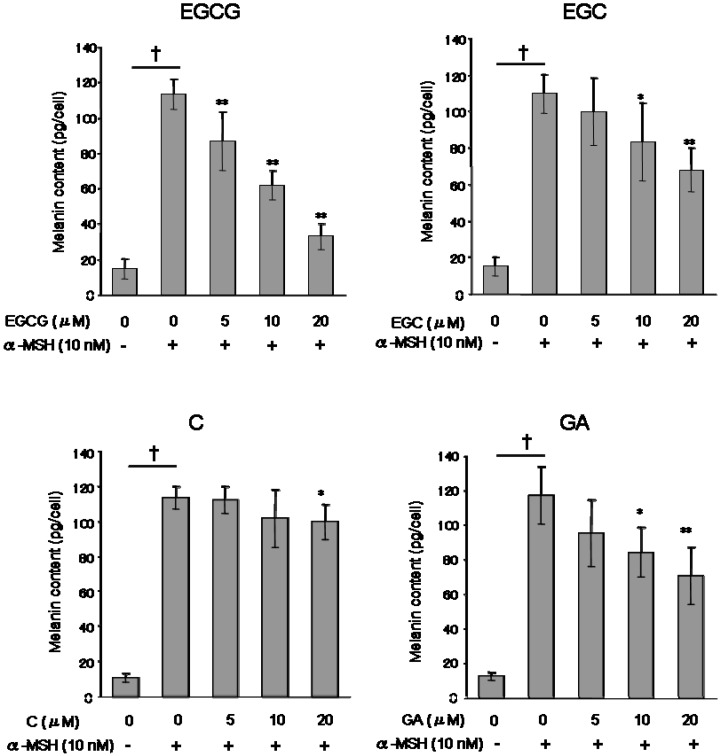
Effect of catechins (EGCG, EGC and C) and GA on melanin production. The melanin content of B16 melanoma cells was measure after 5 days of treatment with or without test substances, as described in the Experimental. Results are expressed as pg/cell, and average of melanin contents per cell of control groups were 113.5 pg/cell (EGCG), 109.4 pg/cell (EGC), 115.3 (C) and 117.4 pg/cell (GA). Data reported are the means ± S.D. of at least three separate experiments. *P < 0.05 and **P < 0.01 versus control (α-MSH alone treated) group. † P < 0.01 versus media only treated group.

To determine the effects of catechins on melanogenesis, B16 melanoma cells stimulated with α-MSH (α-melanocyte stimulating hormone) were cultured in the presence of the catechin group at 5-20 μM for five days. The melanin synthesis of the B16 melanoma cells were stimulated by α-MSH treatment (Figure 3). Treatment with EGCG produced dose-dependent inhibition of melanin formation in B16 melanoma cells stimulated with α-MSH (approximately, 87 pg/cell at 5 μM, 62 pg/cell at 10 μM, and 33 pg/cell at 20 μM). Interestingly, GA inhibited melanogenesis more so than C treatment. 

**Figure 4:**
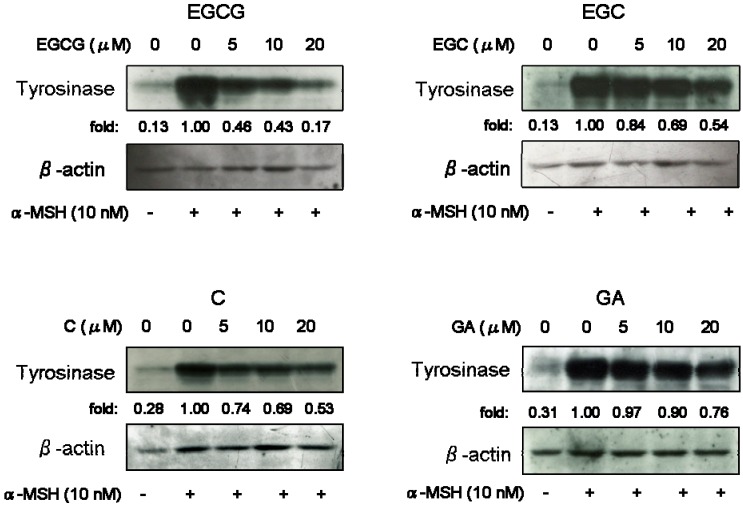
Effects on tyrosinase expression of catechins (EGCG, EGC and C) and GA. B16 melanoma cells were treated with or without test substances and stimulated with α-MSH (nM). After five days treatment, Western blotting of tyrosinase was performed as described in the Experimental. The loading control was assessed using anti-β-actin antibody. Fold increases over the control were determined by densitometric analysis.

All test substances inhibited melanin production (Figure 3). Next, we performed Western blotting analysis to investigate whether catechins and GA can influence tyrosinase protein level. As shown as Figure 4, when cells were stimulated by α-MSH alone, a significant increase of tyrosinase protein was observed. All test substances inhibited α-MSH-stimulated tyrosinase expression of B16 melanoma cells in a dose-dependent manner. Especially, EGCG significantly inhibited tyrosinase level, as did EGC and GA treatment. 

## Conclusion

In this study, we investigated the inhibitory effect of catechins on melanogenesis. All test substances including GA inhibited melanin synthesis of B16 melanoma. Our results show pyrogallol-containing compounds such as EGCG, EGC, and GA have significant inhibitory activity of melanin synthesis. No *et al*. indicated that flavan-3-ols containing a gallic acid moiety at the 3 position shows storong inhibition of tyrosinase activity [15]. Therefore, the depigmenting effect of catechins is due to direct inhibition of tyrosinase activity and down-regulation of tyrosinase expression of B16 melanoma cells. Further studies on the depigmenting mechanism of catechins are now underway. Catechin derivatives are useful inhibitors of melanogenesis and they might lead to an effective treatment for hyperpigmentation disorders.

## Experimental

### Cell culture

B16F1 murine melanoma cells were cultured in Dulbecco’s modified Eagle’s medium (DMEM) (Sigma, St. Louis, MO, USA) supplemented with 5% heat-inactivated fetal bovine serum (FBS), 50 U/mL penicillin, and 50 μg/ml streptomycin at 37℃ in a humidified atmosphere containing 5% CO_2_.

### Cell proliferation

EGCG, EGC, C, and GA (Wako Chemical industries, Ltd) were dissolved in MilliQ water at a stock concentration. These chemicals were added to the cell culture media at a final concentration of 5, 10, and 20 μM, and the culture dishes had α-MSH (Sigma) added to a final concentration of 10 nM. B16 melanoma cells were treated with tocopherol for five days. After treatment, the cells were collected using trypsin/EDTA and were counted with a Fuchs-Rosenthal cytometer.

### Measurement of melanin content

Melanin content was measured as described previously [16]. The cells were treated with EGCG, EGC, C, and GA (5-20 μM) and co-cultured with α-MSH (10 nM) for 5 days. After treatment, the cells were detached after a short incubation with trypsin/EDTA. After precipitation, cell pellets containing a known number of cells were solubilized in boiling 2 M NaOH for 20 min. Spectrophotometric analysis of their melanin content was performed by measuring their absorbance at 405 nm. The absorbance of melanin-containing solution was compared with standard curve of known concentrations of synthetic melanin (Sigma). 

### Western blotting

B16 melanoma cells that had been stimulated by α-MSH were treated with EGCG, EGC, C, and GA for 5 days. After treatment, the cells were collected and lysed in cell lysis buffer (50 mM Tris-HCl [pH 6.8], 2% SDS, 6% β-mercaptoethanol, and 1% glycerol). Whole cell lysates (5 × 10^4 ^cells equivalents per lane) were separated by 7.5% SDS-polyacrylamide gel electrophoresis and transferred to a nitrocellulose membrane. The membranes were blocked by 5% skimmed milk in phosphate-buffered saline containing 0.05% Tween20. Tyrosinase was detected with rabbit polyclonal anti-tyrosinase antibody (dilution 1:1000), which was purchased from Santa Cruz Biotechnology (Santa Cruz, CA, USA). Finally, the membranes were incubated with horseradish peroxidase-conjugated secondary anti-rabbit IgG antibody at a 1:1500 dilution.Bound antibodies were detected using an enhanced chemiluminescence kit (Amersham) according to the manufacturer’s instructions. The intensity of the bands was quantified by the ImageJ program (NIH image, Bethesda, MD, USA) and normalized to the amount of β-actin.

### Statistical analysis

All statistical analysis was carried out using Dunnett’s test. 
